# An Easy to Miss, but Preventable Tragedy: Vasa Previa

**DOI:** 10.1055/s-0040-1713914

**Published:** 2020-09-08

**Authors:** Catarina Reis-de-Carvalho, Maria Afonso, Rui Marques Carvalho

**Affiliations:** 1 Department of Obstetrics, Gynaecology and Reproductive Medicine, Centro Hospitalar Universitário Lisboa Norte, Lisbon, Portugal; 2 Faculty of Medicine, Universidade de Lisboa, Lisbon, Portugal

Dear Editor,

The role of renowned scientific journals, like yours, is to bring innovation, but also education. And so, we decided to bring to your attention a condition that is rare, but still frequent enough to be known to all obstetrician-gynecologists. It is in our hands this simple diagnosis that can prevent an obstetric tragedy.

Vasa previa is defined as the presence of aberrant fetal vessels running within the membranes near the internal os of the cervix as a result of abnormal placentation. 1 There are two types, depending on if the free vessel is connected to a velamentous cord (type I) or connected to a succenturiate or accessory lobe of the main placenta (type II). 2 The estimated incidence is 1 in 2,500 deliveries, but it is much higher (1 in 700 births) among patients who conceive through assisted reproductive technologies. Most cases (85%) have ≥ 1 identifiable risk factors, including in-vitro fertilization, multiple gestations, bilobed, succenturiate or low-lying placentas, and velamentous cord insertion. 3

When the condition is not diagnosed antenatally, the perinatal mortality rate is reported to be ∼ 44%. 3 If an emergency cesarean delivery is required, and if the diagnosis of vasa previa is not made in the antenatal period, < 50% of neonates survive. 4

There is much controversy regarding the screening of vasa previa. Most authors do not recommend universal screening. 5 However, there is a consensus that all cases with recognized risk factors should be routinely screened, ideally around mid-gestation (18–26 weeks), through transvaginal ultrasound with color Doppler imaging (CDI). 1 Ruiter et al 5 identified a median prenatal detection rate of 93% and a specificity of 99% when this approach is taken. Nevertheless, some precautions must be considered to rule out a diagnosis of a pseudo vasa previa, namely, confirm the placental cord insertion and reject the presence of a space between the placental vessels and the internal os. 6

The optimal surveillance strategy in the case of vasa previa, including the need for antepartum hospitalization, is not well-defined. International guidelines recommended a scheduled cesarean section of all asymptomatic women presenting with vasa previa between 34 and 36 weeks of gestation. 2

To illustrate this condition, we would like to portray a case of a 32-year-old primiparous pregnant woman with a diagnosis of anterior low-lying placenta on her routine transabdominal ultrasound at 28 weeks of pregnancy. A transvaginal ultrasound measured a distance of the placental edge to the internal os of 18 mm ( Fig. 1 ), which does not prevent a vaginal delivery. However, the complementary study with the use of CDI allowed the observation of a posterior placental cotyledon (placenta succenturiata), that was 1.5 mm from the internal os, with vessels connecting the two parts of the placenta, running over the internal os (vasa previa) ( Fig. 2 ). Moreover, we found a short cervix with 19 mm of length. Some authors have suggested that there is a relation between a cervical length of ≤ 30 mm, or ≤ 25 mm in other studies, and higher rates of antepartum bleeding requiring emergency delivery. 7 8 Considering the maternal and fetal risks associated, the obstetrician team decided to admit the pregnant women for hospital surveillance and fetal lung maturation. At 33 weeks of gestation, abundant vaginal bleeding occurred, which led to an emergency cesarean section. The newborn was born perfectly well. The mother had mild anemia 48 hours after delivery (Hb 10.1 g/dL) and was treated with oral iron supplementation. Both mother (after 3 days) and baby (after 16 days) were discharged without further complications. The anatomopathological study confirmed the diagnosis of a type 2 vasa previa.

**Fig. 1:**
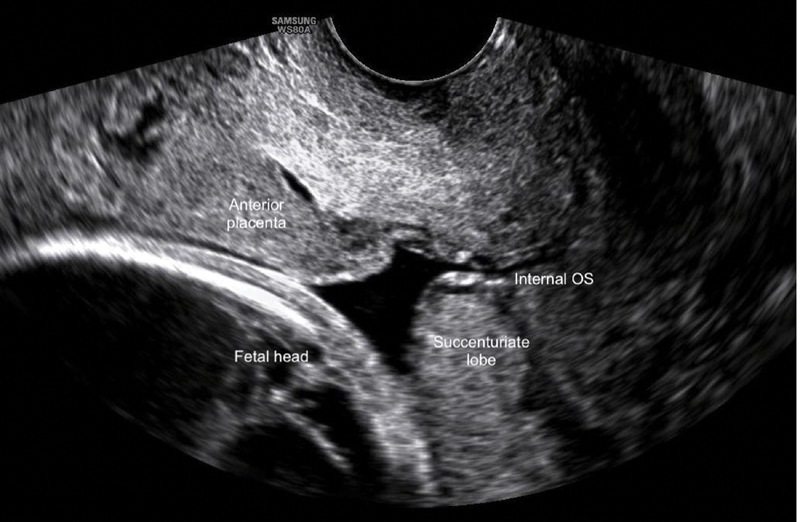
Sagittal section of the cervix, through transvaginal ultrasound, revealing an anterior low-lying placenta and a posterior succenturiate lobe.

**Fig. 2:**
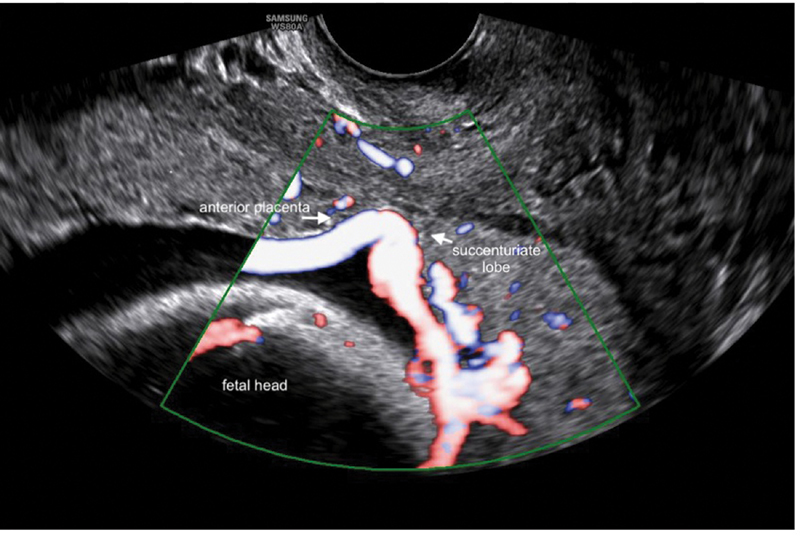
Use of color Doppler allowed the visualization of a vascular structure (vasa previa) that crosses the internal orifice of the cervix, connecting the separate succenturiate lobe to the main portion of the placenta. The arrows indicate the placental limits of both lobules closest to the internal os.

Through this case, it is possible to understand the crucial importance of a timely diagnosis of vasa previa. If we had not made this diagnosis antenatally, our patient wouldn't have been under our close surveillance in the hospital, and the decision for an emergency cesarean section would not have been made so promptly. Because fetal bleeding and death can occur within minutes, any delay in managing this situation would be fatal.

In conclusion, the antenatal screening of vasa previa applying transvaginal ultrasound and color Doppler technology during the obstetrical ultrasound is imperative, especially in cases in which some risk factor is recognized.

## References

[1] Datta S Babu K M Mitra S Patil D Vasa previa: an avoidable obstetric tragedy J Obstet Gynaecol India 2016 66 03 185 187. Doi: 10.1007/s13224-015-0751-4 27298529 10.1007/s13224-015-0751-4 PMC4870668

[2] Melcer Y Maymon R Jauniaux E Vasa previa: prenatal diagnosis and management Curr Opin Obstet Gynecol 2018 30 06 385 391. Doi: 10.1097/GCO.0000000000000478 30102606 10.1097/GCO.0000000000000478

[3] Krief D Naepels P Chevreau J Per labor Vasa Previa discovery: A simple clinical diagnosis Eur J Obstet Gynecol Reprod Biol 2018 231 284 285. Doi: 10.1016/j.ejogrb.2018.10.056 30396705 10.1016/j.ejogrb.2018.10.056

[4] Obstetrix Collaborative Research Network Swank M L Garite T J Maurel K Das A Perlow J H Combs C A Vasa previa: diagnosis and management Am J Obstet Gynecol 2016 215 02 2230 2.23E8. Doi: 10.1016/j.ajog.2016.02.044 10.1016/j.ajog.2016.02.044 26944186

[5] Ruiter L Kok N Limpens J Derks J B de Graaf I M Mol B WJ Pajkrt E Systematic review of accuracy of ultrasound in the diagnosis of vasa previa Ultrasound Obstet Gynecol 2015 45 05 516 522. Doi: 10.1002/uog.14752 25491755 10.1002/uog.14752

[6] Kajimoto E Matsuzaki S Matsuzaki S Tanaka Y Kinugasa-Taniguchi Y Mimura T Challenges in diagnosis of pseudo vasa previa Case Rep Obstet Gynecol 2014 2014 903920. Doi: 10.1155/2014/903920 24971183 10.1155/2014/903920 PMC4058268

[7] Maymon R Melcer Y Tovbin J Pekar-Zlotin M Smorgick N Jauniaux E The rate of cervical length shortening in the management of vasa previa J Ultrasound Med 2018 37 03 717 723. Doi: 10.1002/jum.14411 28880409 10.1002/jum.14411

[8] Oyelese Y Smulian J C Placenta previa, placenta accreta, and vasa previa Obstet Gynecol 2006 107 04 927 941. Doi: 10.1097/01.AOG.0000207559.15715.98 16582134 10.1097/01.AOG.0000207559.15715.98

